# Iran is falling behind WHO cervical cancer elimination targets: HPV vaccination coverage and cervical cancer screening participation in 2021

**DOI:** 10.1371/journal.pone.0341888

**Published:** 2026-02-19

**Authors:** Mohammad Haddadi, Ali Golestani, Yosef Farzi, Nazila Rezaei, Erfan Ghasemi, Sina Azadnajafabad, Negar Rezaei, Naser Ahmadi, Mohammad-Mahdi Rashidi, Maryam Nasserinejad, Ameneh Kazemi, Moein Yoosefi, Elmira Foroutan Mehr, Rosa Haghshenas, Amirali Hajebi, Sahar Mohammadi Fateh, Niusha Nazari, Azadeh Momen Nia Rankohi, Shirin Djalalinia, Farshad Farzadfar

**Affiliations:** 1 Non-Communicable Diseases Research Center, Endocrinology and Metabolism Population Sciences Institute, Tehran University of Medical Sciences, Tehran, Iran; 2 Vali-e-Asr Reproductive Health Research Center, Family Health Research Institute, Tehran University of Medical Sciences, Tehran, Iran; 3 Center for Life Course Health Research, Faculty of Medicine, University of Oulu, Oulu, Finland; 4 Department of Mathematics and Statistics, Memorial University of Newfoundland, St. John’s, Newfoundland and Labrador, Canada; 5 Endocrinology and Metabolism Research Center, Endocrinology and Metabolism Clinical Sciences Institute, Tehran University of Medical Sciences, Tehran, Iran; 6 Development of Research and Technology Center, Deputy of Research and Technology Ministry of Health and Medical Education, Tehran, Iran; University of Nairobi, KENYA

## Abstract

**Objective:**

This study aimed to assess HPV vaccination coverage and cervical cancer screening participation among Iranian females under 46 years old, comparing Iran’s situation to he Cervical Cancer Elimination Initiative (CCEI) targets.

**Methods:**

This nationally and sub-nationally representative cross-sectional study analyzed data from the STEPS 2021 survey. Female participants aged 18–45 years without missing data on HPV vaccination or cervical cancer screening were included. Categorical data were presented as weighted percentages with 95% confidence intervals (95% CI). Logistic regression assessed associations between demographic and female cancer screening variables with the outcomes.

**Results:**

A total of 8,158 females were included. Only 0.85% (95% CI: 0.69–1.02) of women received the HPV vaccine, while cervical cancer screening participation was 39.4% (95% CI: 38.21–40.6). No significant differences in HPV vaccination coverage were observed across age groups. However, screening rates were significantly higher in older women, rising from 27.99% (18–35 years) to 54.07% (36–45 years). HPV vaccination was not significantly associated with demographic variables. In contrast, cervical cancer screening participation was higher among unemployed women (40.58%), married women (49.6%), and those in the highest wealth quintile (42.47% compared to 28.29% in the lowest quintile).

**Conclusion:**

HPV vaccination coverage in Iran is critically low, falling far short of the CCEI target of 90%. Cervical cancer screening participation is comparatively better but still lags approximately 30% behind the target. Strategic interventions are critical to bridge the gap between Iran’s current status and the CCEI targets.

## Introduction

Cervical cancer caused approximately 350,000 deaths among women in 2022, making it the fourth leading cause of cancer-related mortality worldwide [1,2]. Over 85% of cases occur among young, undereducated women from the poorest countries, and 90% of deaths are reported in low- and middle-income countries (LMICs) [3]. This underscores cervical cancer as one of the few diseases marked by significant global health inequalities. Human papillomavirus (HPV), a common sexually transmitted infection (STI), is responsible for 90% of cervical cancers [1,4]. Since 2006, the highly effective HPV vaccine Gardasil has been available in the USA and has become more accessible in many countries [5,6]. Moreover, cervical cancer is highly curable if detected early through screening and timely treatment of pre-cancerous lesions or referral for advanced care when invasive cancer is suspected [7]. Thus, eliminating cervical cancer is achievable by enhancing HPV vaccination coverage, strengthening cervical cancer screening programs, and improving systems for timely management and treatment.

In November 2020, the Cervical Cancer Elimination Initiative (CCEI) was introduced as a comprehensive global strategy aimed at eliminating cervical cancer as a public health concern. The initiative aspires to reduce the incidence of cervical cancer to fewer than four cases per 100,000 women-years worldwide. To achieve this, it sets benchmarks: vaccinating 90% of girls against HPV by age 15, screening 70% of women by age 35 and again by age 45, and ensuring 90% of diagnosed women receive appropriate treatment and management [8]. Although 194 countries have committed to these goals, disparities persist in reaching them, highlighting the need for country-specific evaluations to inform evidence-based decisions [7].

Iran has a unique situation regarding cervical cancer. While the incidence is low and stable at approximately 2.6 cases per 100,000 females, cervical cancer remains more fatal than other gynecological cancers. The mortality-to-incidence ratio (MIR) of cervical cancer declined by 36% from 1990 to 2016, reflecting some progress [9]. This reduction may be attributed to the cervical cancer screening program, which has been in place since the 1980s and primarily relies on Pap smear tests as the standard method [10,11]. However, Iran currently lacks a nationwide HPV vaccination program as a primary prevention strategy [5]. Although the domestically manufactured bivalent vaccine Papilloguard, which targets HPV types 16 and 18, was approved by the Iranian Food and Drug Administration in 2020 [5], it has not been incorporated into the national immunization schedule and remains available only in limited private-sector settings. Multiple sociocultural, economic, and policy-related barriers have hindered its implementation. These include public misconceptions about HPV as a sexually transmitted infection leading to social stigma, limited sexual health education in schools, religious and cultural sensitivities surrounding adolescent vaccination, vaccine cost and lack of insurance coverage, and insufficient governmental prioritization of HPV vaccination within national health policies [12]. Evaluating Iran’s progress toward the CCEI targets could highlight the efficacy of its cervical cancer screening program and underscore the need for establishing a nationwide HPV vaccination program.

Compared to other LMICs, Iran’s HPV vaccination coverage and programmatic readiness remain considerably limited. Several LMICs such as Rwanda have successfully implemented national HPV vaccination programs with coverage rates exceeding 93.23%, largely supported by international initiatives like Gavi, the Vaccine Alliance [13]. In contrast, many middle-income countries, including Iran, have not benefited from such external funding and face additional barriers related to vaccine affordability, cultural acceptance, and health policy prioritization. This places Iran behind global and regional peers in achieving the World Health Organization’s (WHO) CCEI targets.

In this study, we aimed to assess Iran’s progress toward achieving the CCEI targets. We utilized data from the latest WHO STEPwise approach to Non-Communicable Diseases Risk Factor Surveillance (STEPS) 2021 study in Iran, which provides the only nationally representative data on HPV vaccination. We assessed HPV vaccination coverage and cervical cancer screening participation prevalence among adult females under 46 years old. The results of this study, representing the national situation in Iran, could provide valuable insights for developing evidence-based strategies to improve accessibility, equity, and uptake of preventive services, thereby aligning Iran’s efforts with international goals for cervical cancer elimination.

## Materials and methods

### Study design and participants

The STEPS 2021 survey was conducted in Iran as a nationally and sub-nationally representative cross-sectional study, encompassing both rural and urban areas. The study protocol has been previously published [14]. Briefly, a systematic cluster sampling method was used to calculate the sample size based on each province’s population and relative weighting. Initially, 3,167 clusters, each consisting of 10 participants, were planned for sampling. Data collection began in January 2020; however, after completing 318 clusters, the survey was halted due to the onset of the COVID-19 pandemic. Following modifications to the study protocol necessitated by the pandemic, the remaining 2,849 clusters were adjusted to include 9 participants each. Data collection resumed in January 2021 and was completed by the end of April 2021. A limited number of questionnaires were also administered as a pilot in the autumn of 2020.

**Fig 1:**
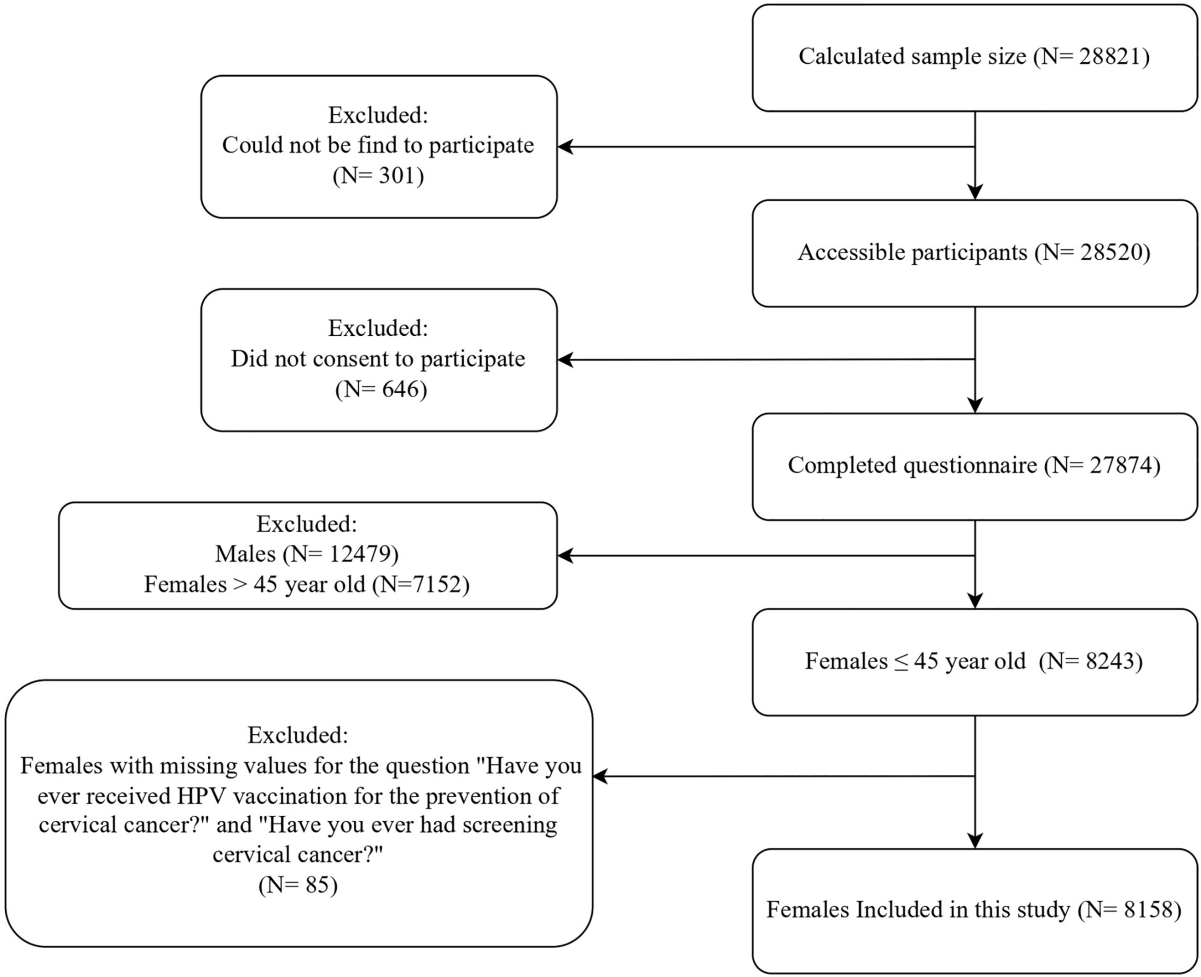
The Study Flowchart.

While the estimated sample size was 28,821 individuals, 27,874 participants ultimately completed the questionnaire. The study included Iranian adults aged ≥18 years. Exclusion criteria were individuals with psychological problems that prevented them from completing the questionnaire, those unable to undergo anthropometric measurements due to physical limitations, individuals for whom laboratory measurements were not possible, and pregnant women. The survey consisted of three steps: (1) data collection using a questionnaire, (2) anthropometric measurements, and (3) biochemical measurements. For this study, only data from the first step (the questionnaire) were utilized. We included females aged ≤45 years who had no missing values for at least one of outcome variables of the study: receipt of the HPV vaccination and cervical cancer screening experience (Fig 1). The data were accessed for research purposes on May 2024 after obtaining all ethical and research committee approvals.

### Variable definition

For this study, we utilized demographic variables and those related to cervical cancer. The demographic features included age, residency (urban/rural), marital status (never married, married, divorced/widowed), employment status (employed/unemployed), province, and health insurance status (none, basic, or basic with complementary). Education was defined as the number of successfully completed years of schooling and categorized into three groups: 0–6 years, 7–12 years, and ≥12 years. The wealth index of each participant was calculated using principal component analysis (PCA) based on household asset data. Participants were then categorized into five wealth quintiles, ranging from the poorest (quintile 1) to the richest (quintile 5).

For HPV vaccination, participants were asked: “Have you ever received the HPV vaccination to prevent cervical cancer?” For cervical cancer screening, the question was: “Have you ever undergone a Pap smear or Human Papilloma Virus (HPV) test?” Additionally, for breast cancer screening, participants were asked: “Have you ever undergone a mammography?”

Age was categorized into four groups (18–20, 21–26, 27–35, and 36–45 years) based on the World Health Organization’s HPV vaccination guidelines (which recommend vaccination for girls aged 9–20 years and catch-up vaccination up to age 26 [15] and CCEI target screening targets (screening at ages 35 and 45 years) [8].

### Statistical methods

A weighting process was conducted to make the analyses representative of national and subnational populations. In this procedure, the calculation of the weights was performed using the general non-responsiveness of participants, non-responsiveness for each step of the study, and age, sex, and area stratified population at the province level based on the 2016 National Population and Housing Census [14]. Categorical data were presented as weighted percentages with 95% confidence intervals (95% CI) calculated using the logit method. The significance of associations was analyzed by the Chi-Square test with adjusted Wald statistic. To determine the association of various variables, two logistic regression models were employed for cervical cancer screening and HPV vaccination statutes. Initially, a univariate regression model was employed to evaluate the association between each independent variable and the dependent outcome, resulting in crude odds ratios (OR). Following this, multiple logistic regression models were applied to adjust for potential confounding variables and reporting adjusted OR (aOR). The adjusted model comprised those variables that had a p-value of less than 0.2 in the univariate analysis [16]. Analysis was conducted in the ‘survey’ library in R software (4.2.3), with statistical significance set at a p-value less than 0.05.

### Ethical considerations

This study was approved by the ethical committee of the Tehran University of Medical Sciences (ID: IR.TUMS.EMRI.REC.1402.119) on 4^th^ February 2024. Written informed consent was obtained from all participants. This study was performed according to Helsinki deceleration and the participant’s identity is confidential [17].

## Results

Overall, 8,158 women aged 18–45 years were included in the study. The majority of participants belonged to the 36–45-year-old age group (43.74%) and resided in urban areas (73.69%). Additionally, 58.82% of participants had more than 12 years of education, 82.71% were unemployed, and 73.95% were married. Health insurance coverage was reported by 69.63% of participants with basic insurance only and by 19.44% with both basic and complementary insurance (Table 1).

**Table 1:** The baseline characteristic of the participants and the prevalence of HPV vaccination coverage and cervical cancer screening prevalence.

Variable		Total (Number; weighted prevalence (95% CI))	HPV vaccination coverage (Weighted prevalence (95% CI))		p-value	Cervical cancer screening statues (Weighted prevalence (95% CI))		p-value
			No	Yes		No	Yes	
Total			99.15 (98.88-99.35)	0.85 (0.69-1.02)	60.6 (59.4-61.79)	39.4 (38.21-40.6)
Age group	18-20	651 (7.72; 7.11-8.38)	99.98.41-99.91)	0.37 (0.09-1.59)	0.294	96.35 (94.34-97.66)	3.65 (2.34-5.66)	<0.001
	21-26	1177 (14.4; 13.58-15.27)	98.8 (97.75-99.36)	1.2 (0.64-2.25)		84.98 (82.52-87.14)	15.02 (12.86-17.48)	
	27-35	2830 (34.14; 33-35.3)	99.1 (98.59-99.43)	0.9 (0.57-1.41)		61.08 (59.03-63.1)	38.92 (36.9-40.97)	
	36-45	3500 (43.74; 42.54-44.95)	99.22 (98.81-99.49)	0.78 (0.51-1.19)		45.93 (44.09-47.78)	54.07 (52.22-55.91)	
Residential area	rural	2372 (26.31; 25.28-27.36)	99.3 (98.76-99.6)	0.7 (0.4-1.24)	0.423	62.72 (60.48-64.9)	37.28 (35.1-39.52)	0.032
	urban	5786 (73.69; 72.64-74.72)	99.1 (98.77-99.34)	0.9 (0.66-1.23)		59.85 (58.43-61.26)	40.15 (38.74-41.57)	
Years of schooling	0-6 years	1834 (20.95; 20-21.93)	99.32 (98.7-99.65)	0.68 (0.35-1.3)	0.581	55.83 (53.24-58.38)	44.17 (41.62-46.76)	<0.001
	7-12 years	1651 (20.23; 19.28-21.23)	99.24 (98.58-99.6)	0.76 (0.4-1.42)		56.28 (53.58-58.94)	43.72 (41.06-46.42)	
	>12 years	4615 (58.82; 57.62-60)	99.05 (98.66-99.32)	0.95 (0.68-1.34)		63.54 (61.96-65.09)	36.46 (34.91-38.04)	
Employment status	Unemployed	6767 (82.71; 81.76-83.63)	99.19 (98.9-99.41)	0.81 (0.59-1.1)	0.429	59.42 (58.09-60.73)	40.58 (39.27-41.91)	<0.001
	Employed	1333 (17.29; 16.37-18.24)	98.91 (98.03-99.4)	1.09 (0.6-1.97)		65.41 (62.47-68.23)	34.59 (31.77-37.53)	
Marriage status	Never married	1771 (21.37; 20.4-22.38)	99 (98.24-99.43)	1 (0.57-1.76)	0.673	96.88 (95.79-97.7)	3.12 (2.3-4.21)	<0.001
	Married	6024 (73.95; 72.88-75)	99.22 (98.91-99.43)	0.78 (0.57-1.09)		50.4 (48.98-51.82)	49.6 (48.18-51.02)	
	Divorced/Widow	363 (4.67; 4.18-5.21)	98.81 (96.56-99.59)	1.19 (0.41-3.44)		56.2 (50.5-61.73)	43.8 (38.27-49.5)	
Wealth index quintiles	1^st^ (poorest)	1365 (17.67; 16.74-18.65)	99.02 (98.2-99.47)	0.98 (0.53-1.8)	0.461	71.71 (68.94-74.33)	28.29 (25.67-31.06)	<0.001
	2^nd^	1462 (20.44; 19.42-21.49)	98.93 (98.08-99.41)	1.07 (0.59-1.92)		64.3 (61.51-67.01)	35.7 (32.99-38.49)	
	3^rd^	1493 (19.19; 18.21-20.21)	99.5 (98.89-99.77)	0.5 (0.23-1.11)		59.46 (56.57-62.28)	40.54 (37.72-43.43)	
	4^th^	1500 (20.29; 19.28-21.33)	99.22 (98.49-99.6)	0.78 (0.4-1.51)		54.99 (52.14-57.81)	45.01 (42.19-47.86)	
	5^th^ (wealthiest)	1552 (22.41; 21.35-23.51)	99.01 (98.25-99.45)	0.99 (0.55-1.75)		57.53 (54.78-60.23)	42.47 (39.77-45.22)	
Health insurance	No	811 (10.93; 10.17-11.73)	98.97 (97.88-99.51)	1.03 (0.49-2.12)	0.784	63.22 (59.49-66.8)	36.78 (33.2-40.51)	<0.001
	Basic	5710 (69.63; 68.49-70.75)	99.12 (98.79-99.37)	0.88 (0.63-1.21)		61.57 (60.14-62.99)	38.43 (37.01-39.86)	
	Basic + Complementary	1537 (19.44; 18.49-20.44)	99.28 (98.53-99.65)	0.72 (0.35-1.47)		54.75 (51.93-57.54)	45.25 (42.46-48.07)	
Cervical Cancer Screening	No	4976 (60.6; 59.4-61.79)	99.43 (99.12-99.63)	0.57 (0.37-0.88)	0.006	–	–	–
	Yes	3146 (39.4; 38.21-40.6)	98.71 (98.16-99.09)	1.29 (0.91-1.84)		–	–	
Mammography	No	6872 (84.41; 83.5-85.28)	99.31 (99.05-99.51)	0.69 (0.49-0.95)	0.015	67.59 (66.33-68.83)	32.41 (31.17-33.67)	<0.001
	Yes	1237 (15.59; 14.72-16.5)	98.2 (97.08-98.89)	1.8 (1.11-2.92)		22.34 (19.85-25.06)	77.66 (74.94-80.15)	
HPV vaccination	No	7910 (99.15; 98.88-99.35)	–	–	–	61 (59.78-62.2)	39 (37.8-40.22)	0.006
	Yes	64 (0.85; 0.65-1.12)	–	–		40.69 (28-54.76)	59.31 (45.24-72)	

95% CI = 95% confidence interval.

The overall HPV vaccination coverage was 0.85% (95% CI: 0.69–1.02). No significant differences in HPV vaccination coverage were observed across subcategories of age, residential area, years of schooling, employment status, marital status, wealth index, or health insurance, and all were not significantly different to the overall HPV vaccination coverage. However, significant differences were found among participants who had undergone cervical cancer screening and mammography, with HPV vaccination coverage of 1.29% (0.91–1.84) and 1.8% (1.11–2.92), respectively (Table 1). Logistic regression analysis showed that undergoing cervical cancer screening (aOR = 2.22 [95% CI: 1.15–4.29]) and mammography (aOR = 2.4 [1.25–4.58]) were significantly associated with receiving the HPV vaccine. No significant associations were observed for other variables in the adjusted models (Table 2).

**Table 2:** Association of different variables with HPV vaccination.

variables		Crude Model		Adjusted Model^*^	
		OR (CI95%)	P-value	OR (CI95%)	p-value
Age group	18-20	Reference	Reference	Reference	Reference
	21-26	3.27 (0.66-16.13)	0.146	2.36 (0.47-11.78)	0.295
	27-35	2.43 (0.52-11.29)	0.256	1.37 (0.29-6.53)	0.69
	36-45	2.11 (0.46-9.7)	0.338	0.9 (0.18-4.52)	0.896
Residential area	Rural	Reference	Reference	–	–
	Urban	1.29 (0.67-2.48)	0.45	–	–
Years of schooling	0-6 years	Reference	Reference	–	–
	6-12 years	1.12 (0.45-2.78)	0.81	–	–
	. > 12 years	1.41 (0.67-2.95)	0.365	–	–
Employment status	Unemployed	Reference	Reference	–	–
	Employed	1.35 (0.69-2.65)	0.385	--	--
Marriage status	Never married	Reference	Reference	–	–
	Married	0.78 (0.41-1.5)	0.458	–	–
	Divorced/widowed	1.19 (0.35-4.03)	0.782	–	–
Wealth index quintiles	1^st^ (poorest)	Reference	Reference	Reference	Reference
	2^nd^	1.09 (0.46-2.56)	0.844	1 (0.43-2.32)	0.991
	3^rd^	0.51 (0.19-1.39)	0.189	0.45 (0.17-1.2)	0.111
	4^th^	0.79 (0.32-1.96)	0.616	0.66 (0.27-1.62)	0.364
	5^th^ (wealthiest)	1.01 (0.43-2.34)	0.989	0.82 (0.35-1.92)	0.65
Cervical cancer screening	No	Reference	Reference	Reference	Reference
	Yes	2.28 (1.3-3.99)	0.004	**2.22 (1.15-4.29)**	**0.018**
Mammography	No	Reference	Reference	Reference	Reference
	Yes	2.66 (1.47-4.81)	0.001	**2.4 (1.25-4.58)**	**0.008**

The logistic regression was used for all analysis.

P-value<0.05 is considered statistically significant.

Variables with p-value <0.2 in univariate analysis were considered for adjusted model.

In contrast to HPV vaccination coverage, significant variation in cervical cancer screening participation was observed across different subcategories of variables. Overall, the prevalence of cervical cancer screening participation was 39.4% (95% CI: 38.21–40.6). Screening rates were significantly higher in older women, rising from 27.99% (18–35 years) to 54.07% (36–45 years). Urban residents had a higher prevalence of screening compared to rural residents (40.15% [95% CI: 38.74–41.57] vs. 37.28% [35.1–39.52]). Similarly, unemployed women were more likely to have undergone screening than employed women (40.58% [39.27–41.91] vs. 34.59% [31.77–37.53]). Nearly half of married women (49.6% [48.18–51.02]) reported cervical cancer screening, whereas prevalence was much lower among single women (Table 1).

Cervical cancer screening participation decreased with higher education levels, with 36.46% (95% CI: 34.91–38.04) among women with ≥12 years of schooling compared to 44.17% (41.62–46.76) in those with 0–6 years of schooling. Screening prevalence showed a positive trend across wealth quintiles, increasing from 28.29% (25.67–31.06) in the lowest quintile to 42.47% (39.77–45.22) in the highest. Women with both basic and complementary health insurance reported higher screening participation (45.25% [95% CI: 42.46–48.07]). Screening participation was notably higher among women who had undergone mammography (77.66% [74.94–80.15]) or received the HPV vaccine (59.31% [45.24–72.00]) (Table 1).

While all variables were associated with cervical cancer screening participation in univariate analysis, the adjusted model revealed no significant associations for education or employment status. Marital status showed a strong association, with married women (aOR = 17.51 [95% CI: 12.61–24.32]) and divorced/widowed women (aOR = 14.59 [9.61–22.14]) having significantly higher odds of screening compared to single women. Older age groups were also significantly associated with screening, with women aged 27–35 years (aOR = 2.65 [2.14–3.29]) and 36–45 years (aOR = 23.76 [3.03–4.67]) having higher odds compared to those aged 18–26 years. Being employed was associated with lower odds of screening (aOR = 0.81 [0.67–0.97]). Wealth index quintiles showed a positive trend, with women in the highest quintile having nearly double the odds of screening (aOR = 1.96 [1.57–2.46]) compared to those in the lowest quintile. Women who had undergone mammography (aOR = 5.1 [4.16–6.25]) and those who received the HPV vaccine (aOR = 2.93 [1.23–7.00]) were also more likely to have undergone cervical cancer screening (Table 3).

**Table 3:** Association of different variables with cervical cancer screening.

variables		Crude Model		Adjusted Model^*^	
		OR (CI95%)	P-value	OR (CI95%)	p-value
Age group	18-26	Reference	Reference	Reference	Reference
	27-35	5.12 (4.25-6.17)	<0.0001	**2.65 (2.14-3.29)**	**<0.0001**
	36-45	9.46 (7.89-11.34)	<0.0001	**3.76 (3.03-4.67)**	**<0.0001**
Residential area	Rural	Reference	Reference	Reference	Reference
	Urban	1.13 (1.01-1.26)	0.033	1.03 (0.88-1.19)	0.744
Years of schooling	0-6 years	Reference	Reference	Reference	Reference
	6-12 years	0.98 (0.84-1.14)	0.811	1.13 (0.93-1.37)	0.21
	. > 12 years	0.73 (0.64-0.82)	<0.0001	1.01 (0.84-1.21)	0.911
Employment status	Unemployed	Reference	Reference	Reference	Reference
	Employed	0.77 (0.67-0.89)	<0.001	**0.81 (0.67-0.97)**	**0.023**
Marriage status	Never married	Reference	Reference	Reference	Reference
	Married	30.57 (22.29-41.94)	<0.0001	**17.51 (12.61-24.32)**	**<0.0001**
	Divorced/widowed	24.22 (16.46-35.62)	<0.0001	**14.59 (9.61-22.14)**	**<0.0001**
Wealth index quintiles	1^st^ (poorest)	Reference	Reference	Reference	Reference
	2^nd^	1.41 (1.18-1.68)	<0.0001	**1.35 (1.1-1.67)**	**0.004**
	3^rd^	1.73 (1.45-2.07)	<0.0001	**1.59 (1.29-1.96)**	**<0.0001**
	4^th^	2.08 (1.74-2.47)	<0.0001	**1.97 (1.59-2.45)**	**<0.0001**
	5^th^ (wealthiest)	1.87 (1.57-2.23)	<0.0001	**1.96 (1.57-2.46)**	**<0.0001**
HPV Vaccination	No	Reference	Reference	Reference	Reference
	Yes	2.28 (1.3-3.99)	0.004	**2.93 (1.23-7)**	**0.015**
Mammography	No	Reference	Reference	Reference	Reference
	Yes	7.25 (6.17-8.51)	<0.0001	**5.1 (4.16-6.25)**	**<0.0001**

The logistic regression was used for all analysis.

P-value<0.05 is considered statistically significant.

Variable with p-value <0.2 in univariate analysis were considered for adjusted model.

**Fig 2:**
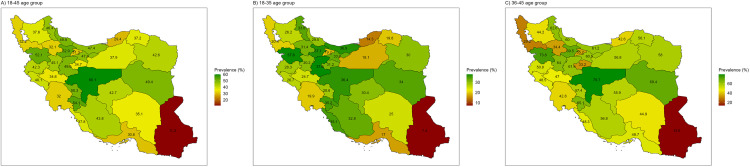
The cervical cancer screening participation in Iran.

The overall prevalence of cervical cancer screening participation among females aged 18–45 years showed substantial variation across provinces in Iran (Fig 2). The prevalence ranged from 60.08% in Isfahan to 30.58% in Hormozgan. Notably, Sistan and Baluchestan province exhibited an outlier low prevalence of 11.23%, significantly lower than other provinces. When considering age groups, the disparity in cervical cancer screening participation was more pronounced among women aged 36–45 years, ranging from 79.74% in Isfahan to 13.58% in Sistan and Baluchestan. In comparison, the disparity was less marked in the 18–35 age group, with prevalence ranging from 37.8% to 7.4%. Detailed prevalences for each province are provided in S1 Table.

## Discussion

This study evaluated HPV vaccination coverage and cervical cancer screening participation among females aged 18–45 years in Iran, using nationally and sub-nationally representative data from STEPS 2021. The HPV vaccination coverage was alarmingly low at 0.85% and showed no significant associations with demographic variables. However, females who had undergone mammography or cervical cancer screening were significantly more likely to have received the HPV vaccine. Cervical cancer screening participation was 39% overall, with prevalence rates of 28% among females aged 18–35 years and 54% among those aged 36–45 years. Screening participation was associated with demographic factors such as age, marital status, employment, and wealth, as well as with undergoing mammography and HPV vaccination. Substantial subnational variations in cervical cancer screening prevalence were observed, with some provinces nearing the CCEI targets, while many remained far from achieving them. Overall, while HPV vaccination coverage is critically low—falling far short of the CCEI target of 90%—the situation for cervical cancer screening is comparatively better, yet still lags approximately 30% behind the target. The low prevalence of cervical cancer screening and HPV vaccination in Sistan and Baluchestan may reflect sociocultural barriers, limited healthcare access, and low awareness. In contrast, higher-prevalence regions such as Isfahan might indicate gaps in policy implementation, resource allocation, or program reach, suggesting the need for targeted interventions. Unexpectedly, our study shows that cervical cancer screening participation decreased with higher education levels. This unexpected pattern may reflect cultural stigma and privacy concerns among more educated women, who might prefer private healthcare settings where routine screening is less emphasized. Additionally, that marriage rates might be lower among individuals with higher education compared to those with lower education [18], which could partly explain the reduced screening participation in this group. However, after adjustment in the multivariate analysis, this association was not statistically significant.

The CCEI recommends that 90% of girls be fully vaccinated with the HPV vaccine by age 15 to achieve primary prevention of cervical cancer. This target is based on evidence showing an 87% reduction in cervical cancer incidence and a 97% reduction in grade 3 cervical intraepithelial neoplasia among women vaccinated at ages 12–13 [7]. The U.S. Food and Drug Administration (FDA) approved the quadrivalent HPV vaccine (Gardasil 4) in 2006 [5], while Iran approved its bivalent HPV vaccine (Papilloguard) in 2020 [19]. Papilloguard provides protection against HPV types 16 and 18, which are responsible for approximately 70% of cervical cancers and 90% of HPV-related malignancies worldwide [20,21]. However, the vaccine is not yet included in Iran’s national immunization schedule, and its cost-effectiveness remains uncertain [10–13]. It is unclear when the Iranian population first gained access to the HPV vaccine before 2020, as no specific date has been documented. HPV vaccination coverage depends on various factors, including income levels, internal or external funding, and other socioeconomic determinants [22]. However, our study found no significant differences in HPV vaccination coverage across different sociodemographic groups in Iran, indicating that all population groups should be considered for targeted interventions.

In this study, we focused on females aged 18–45 years, whereas the CCEI target for HPV vaccination is for girls by the age of 15. Although no national data are available on HPV vaccination coverage for girls under 15 in Iran, our analysis showed no significant differences in vaccination rates among the included age groups or other demographic variables. This consistency suggests that the findings may be generalizable to younger populations, particularly given the lack of a structured HPV vaccination program in Iran. While Iran’s situation in achieving the CCEI target is dire, other countries—both developed and developing—also fall short of the target but perform significantly better than Iran. For instance, approximately 60% of female adolescents aged 13–15 years received the HPV vaccine in the United States during 2021–2022. Similarly, about 58% of Brazilian female adolescents aged 9–15 were vaccinated [23]. A single-center study in Morocco in 2024 reported that 33% of parents of girls eligible for HPV vaccination had vaccinated their daughters [24]. In the United Kingdom, a national program delivers HPV vaccination to girls in years 8 and 9, with an estimated coverage of 71.3% for year 8 females during 2022–2023 [25].

According to the Centers for Disease Control and Prevention (CDC), the HPV vaccine is recommended for children at the age of 11 or 12 but can be administered as early as age 9 and up to age 26 for those who have not yet been vaccinated [26]. The WHO recommends a one- or two-dose schedule for girls and women aged 9–20 and a two-dose schedule with a six-month interval for women aged 21 and older [27,28]. There is also an alternative off-label strategy of single-dose vaccination of 9–20-year-olds [15]. Current evidence suggests that a single dose offers comparable efficacy and duration of protection to a two-dose schedule, potentially improving program efficiency, affordability, and coverage [15].

Despite the global emphasis on HPV vaccination, its implementation in Iran faces challenges due to cost-effectiveness concerns, as well as political and cultural issues [29,30]. The government could benefit from reevaluating the vaccine’s cost-effectiveness, considering its potential long-term advantages in preventing HPV-related cancers and also alternative strategies. A policy revision could include incorporating HPV vaccination into the national immunization schedule, prioritizing girls aged 9–14 in alignment with WHO guidelines [27,28,31], or alternative off-label single dose strategy of 9–20 years old [15]. Additionally, the policy should aim to raise public awareness about the benefits of HPV vaccination and address any misconceptions or hesitations regarding the vaccine.

Beyond cost and policy considerations, vaccine hesitancy in Iran is influenced by economic, cultural, and religious factors. The high out-of-pocket cost of the HPV vaccine, currently not covered by insurance, limits accessibility, especially among low-income families, raising cost-effectiveness concerns from both individual and system-level perspectives [29,30]. Religious and cultural beliefs surrounding premarital sexual activity may also reduce parental acceptance of vaccinating adolescent girls, as HPV is often misperceived as a vaccine promoting sexual permissiveness [32]. Moreover, limited sexual health education could perpetuate misinformation and mistrust regarding the vaccine’s safety and necessity [33]. Studies in other LMICs demonstrate that integrating HPV vaccination within school-based or faith-supported health programs, coupled with transparent communication about its cancer-preventive role rather than its sexual connotations, effectively mitigates hesitancy and improves uptake [34]. For example, Rwanda achieved over 90% coverage through a school-based delivery strategy and strong political commitment, ensuring free access to all eligible girls [13]. This example show that clear communication, community involvement, and government support are key to successful HPV vaccination in LMICs. Similar approaches could help Iran build an affordable and acceptable national HPV program.

Cervical cancer is highly treatable if detected early through screening and timely treatment of pre-cancerous lesions or referral for advanced care when invasive cancer is suspected [7]. The WHO recommends HPV DNA testing as the primary screening method, as it is more effective than visual inspection with acetic acid or cytology. Screening can be conducted using either self-collected or provider-collected cervical samples, following a screen-and-treat or screen-triage-and-treat approach. To ensure inclusivity, the WHO also emphasizes improving screening services for intersex, gender-fluid, and transgender individuals with a cervix [23]. Our study revealed that the prevalence of cervical cancer screening among women aged 36–45 years was 54.07%, approximately 15% below the CCEI target. This higher prevalence compared to HPV vaccination may be attributed to the cervical cancer screening program established in Iran in the 1980s, which primarily relies on Pap smear tests. The long-standing Pap smear program since the 1980s has increased awareness and familiarity with screening, explaining its relatively higher uptake compared with HPV vaccination. Current guidelines recommend that women aged 21–65 years undergo screening every three years if using cytology alone or every five years if combined with HPV DNA testing [10]. Despite this, significant disparities exist across provinces. For example, screening rates in Isfahan and Kordestan approached the CCEI target, while provinces like Sistan and Baluchestan fell significantly short.

Cervical cancer screening coverage remains suboptimal due to cultural barriers, lack of awareness, and limited access to healthcare services, particularly in rural areas [35]. The unavailability of screening facilities, insufficient knowledge about cervical cancer and its screening methods, and socio-cultural factors have been identified as primary barriers to the utilization of cervical cancer screening services among women in African countries [36]. Similarly, in low- and middle-income countries, inadequate awareness about cervical cancer, under-resourced healthcare systems, restricted access to healthcare services, and gender norms that deprioritize women’s health pose significant obstacles to cervical cancer screening [37]. Additionally, studies have shown that women from ethnic minority groups are less likely to participate in cervical screening due to emotional barriers, such as fear, embarrassment, and anticipated shame [38]. Consistent with these findings, our study revealed that being employed, single, or belonging to a lower wealth index was significantly associated with lower cervical cancer screening rates. These results highlight the importance of addressing socioeconomic and cultural barriers to improve screening participation and ensure equitable access to cervical cancer prevention services.

To address the suboptimal cervical cancer screening rates and align with the Cervical Cancer Elimination Initiative (CCEI) targets, a comprehensive policy framework should prioritize improving awareness, accessibility, and integration of services. Community-based awareness campaigns that leverage culturally tailored approaches and diverse media platforms can effectively dispel misconceptions and encourage screening uptake [39]. A key finding of this study was the significant association between undergoing mammography, cervical cancer screening participation, and HPV vaccination. This suggests that individuals who engage in preventive health measures, such as mammography, are more likely to participate in Pap smear screening and HPV vaccination. These results underscore the importance of increasing awareness among women in Iran regarding preventive health measures. Alongside developing structured programs for HPV vaccination and cervical cancer screening, campaigns aimed at enhancing knowledge about cervical cancer could substantially improve progress toward achieving CCEI targets. Moreover, transitioning to HPV testing as the primary screening method, coupled with the availability of self-sampling kits, offers higher sensitivity and greater convenience, particularly in low-resource settings [40]. Strengthening healthcare provider training and referral systems is also essential to ensure timely follow-ups and treatment. Establishing a robust national cancer registry would enable continuous monitoring and evaluation of screening programs [41]. Addressing significant barriers to cervical cancer screening, such as socioeconomic disparities, requires evidence-based strategies informed by expert opinions, consensus-building, and multidisciplinary collaboration. Targeted interventions that address these challenges holistically can effectively mitigate obstacles and advance the nation toward optimal cervical cancer screening coverage.

A key strength of this study is its national and subnational representative sample. However, several limitations should be acknowledged. Notably, data regarding the type and dosage of HPV vaccines administered within the population, as well as the timing of vaccine implementation, were not available. Furthermore, the STEPS study included only individuals aged 18 years and older, thereby excluding data on the current vaccination prevalence among female adolescents. Additionally, information related to the type of cervical cancer screening, the frequency and timing of screenings throughout participants’ lives, and the age at which screenings were initiated was not captured in this study.

## Conclusions

HPV vaccination coverage and cervical cancer screening participation in Iran remain considerably below optimal levels, highlighting the need for a comprehensive strategy that integrates vaccination, screening, and awareness campaigns to achieve the CCEI targets in Iran. The observed disparities in preventive care utilization, particularly across age groups, socioeconomic status, and marital status, emphasize the importance of addressing structural and social determinants of health. Strengthening public health investment in primary prevention, such as subsidizing the HPV vaccine and implementing school-based vaccination programs, can improve affordability, accessibility, and uptake. Enhancing public awareness through culturally sensitive education campaigns and integrating screening and vaccination services into primary healthcare settings are critical steps toward reducing the cervical cancer burden and achieving equitable healthcare outcomes.

## Supporting information

S1 Table Cervical screening in provinces of Iran. (CSV)

## Abbreviations

LMICs: low- and middle-income countries
HPV: human papillomavirus
CCEI: Cervical Cancer Elimination Initiative
MIR: The mortality-to-incidence ratio
STEPS: STEPwise approach to Non-Communicable Diseases Risk Factor Surveillance
CI: confidence intervals
OR: odds ratio
aOR: adjusted OR odds ratio.
